# Identification of core cuprotosis-correlated biomarkers in abdominal aortic aneurysm immune microenvironment based on bioinformatics

**DOI:** 10.3389/fimmu.2023.1138126

**Published:** 2023-04-17

**Authors:** Jiateng Hu, Song Xue, Zhijue Xu, Zhaoyu Wu, Xintong Xu, Xin Wang, Guang Liu, Xinwu Lu, Bo Li, Xiaobing Liu

**Affiliations:** ^1^ Department of Vascular Surgery, Shanghai Ninth People’s Hospital, Shanghai Jiao Tong University School of Medicine, Shanghai, China; ^2^ Vascular Centre of Shanghai Jiao Tong University, Shanghai, China; ^3^ Department of Orthopedics, Ruijin Hospital, Shanghai Jiao Tong University School of Medicine, Shanghai, China

**Keywords:** Abdominal aortic aneurysm, Cuprotosis, FDX1, NLRP3, immune environment

## Abstract

**Background:**

The occurrence of abdominal aortic aneurysms (AAAs) is related to the disorder of immune microenvironment. Cuprotosis was reported to influence the immune microenvironment. The objective of this study is to identify cuprotosis-related genes involved in the pathogenesis and progression of AAA.

**Methods:**

Differentially expressed lncRNAs (DElncRNAs) and mRNAs (DEmRNAs) in mouse were identified following AAA through high-throughput RNA sequencing. The enrichment analyses of pathway were selected through Gene Ontology (GO), Kyoto Encyclopedia of Genes and Genomes (KEGG). The validation of cuprotosis-related genes was conducted through immunofluorescence and western blot analyses.

**Results:**

Totally, 27616 lncRNAs and 2189 mRNAs were observed to be differentially expressed (|Fold Change| ≥ 2 and q< 0.05) after AAA, including 10424 up-regulated and 17192 down-regulated lncRNAs, 1904 up-regulated and 285 down-regulated mRNAs. Gene ontology and KEGG pathway analysis showed that the DElncRNAs and DEmRNAs were implicated in many different biological processes and pathways. Furthermore, Cuprotosis-related genes (NLRP3, FDX1) were upregulated in the AAA samples compared with the normal one.

**Conclusion:**

Cuprotosis-related genes (NLRP3,FDX1) involved in AAA immune environment might be critical for providing new insight into identification of potential targets for AAA therapy.

## Introduction

Abdominal aortic aneurysms (AAAs) exist as a devastating chronic inflammatory vascular disease characterized by segmental and permanent dilation of the abdominal aorta. AAA rupture is often lethal with more than 85% mortality (1). In various studies, lipid peroxidation exerts significant influence on cardiovascular diseases including atherosclerosis and AAA (2, 3). Meanwhile, AAA is the late stage of atherosclerosis (4). Furthermore, inflammation has been demonstrated to play vital role in AAA pathology. Our previous study confirmed that inactivate TXNIP-NLRP3 inflammasome of macrophages helped inhibit the chronic inflammation of aorta thus decreasing the incidence of AAA (5). In addition, one of the key mechanisms is vascular smooth muscle cell (VSMC) apoptosis, which is considered as the critical pathogenesis of the weakness of aorta wall (6, 7). It is noteworthy that apoptosis is an important mode of programmed cell death in the courses of atherosclerosis and AAA. What is more, the widely recognized modes of cell death also include ferroptosis, necroptosis, and autophagy, pyroptosis. Recently, cuprotosis is defined as a novel manner of programmed cell death dependent on copper level, whose effect has been demonstrated in various diseases (8–10).

Copper (Cu) is an essential trace element and catalytic cofactor. Under normal physiological conditions, copper ions maintain a low concentration and dynamic balance in organisms. When copper ions accumulate abnormally, copper toxicity can be induced (11). Specifically, the direct binding of copper to the fatty acylated component of the TCA cycle (TCA) cause the abnormal aggregation of fatty acylated proteins and the loss of iron-sulfur protein thereby leading to protein toxic stress followed by cell death. Recently, the link between cuprotosis and tumor microenvironment (TME) has been reported in various studies (8). And AAA has long been considered as a chronic inflammatory vascular disease, in the formation of which immune microenvironment exerts critical influence (12). Accumulating evidence indicates that the imbalance of copper ion homeostasis in human induces various inflammatory vascular diseases including atherosclerosis and AAA (13). Interestingly, in the progress of some inflammatory diseases, the elevation of lipid oxidation and copper ions in macrophages precedes the activation of inflammatory macrophages and the up-regulation of inflammatory factor (14). Copper ions play a critical role in atherosclerotic plaque formation by influencing lipoprotein metabolism, antioxidant enzymes, low-density lipoprotein oxidation, and inflammation (15). Moreover, available evidence suggests that copper ion levels are significantly elevated in atherosclerotic and AAA tissues under pathological inflammatory conditions (16). A variety of studies have found that copper ion carriers and their chelators are expected to be potential drug molecules for the treatment of inflammatory vascular diseases (13, 17). For instance, the level of cellular copper ions is strictly regulated by copper transporter due to the toxic effect of excessive copper ions (18). Previous studies also found that copper ion transporter ATP7A inhibits the formation of AAA by inhibiting smooth muscle cell apoptosis (16). It is suggested that treating ATP7A with copper chelating agent or regulating the copper ion transport function may be a potential target for treating AAA.

**Figure 1:**
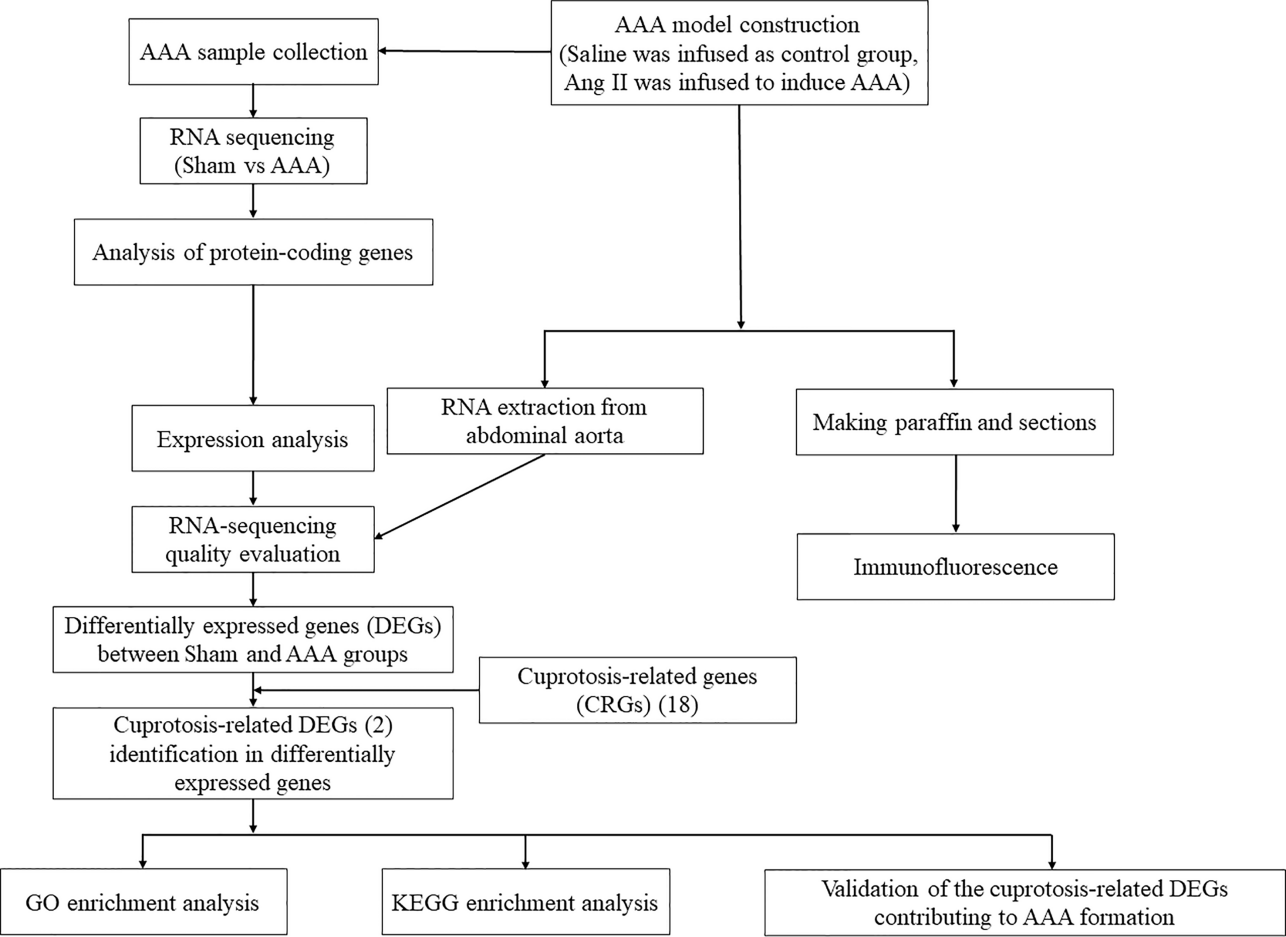
The workflow of this study.

However, the biological role and potential mechanisms of cuprotosis and AAA immune environment has not been fully understood and reported. Meanwhile, based on comprehensive study, it is a promising strategy that we should regulate the expression of cuprotosis-related genes (CRGs) in order to control AAA formation and progression. Hence, we aimed to identify and validate the crucial CRGs involved in the pathological development of AAA, thus revealing the role of cuprotosis in AAA immune environment. In this present study, we intended to explore the transcriptomic profiles and correlation between cuprotosis-related genes through RNA-sequencing analysis, wishing to provide a new strategy for the physiopathologic mechanism and treatment of AAA. An overview of the research was presented in **Figure 1**.

## Materials and methods

### Cell culture and treatment

Aortic vascular smooth muscle cells (VSMCs) (MOVAS-1) were purchased from Guangzhou Geneseed Biotech and were cultured in Dulbecco’s modified Eagle’s medium (DMEM) containing 10% fetal bovine serum (FBS), 100 U/mL penicillin, and 100 μg/mL streptomycin. When VMSCs were cultured to the passage 3 and reached 70-80% confluence, Ang II (1 μmol/L) was applied to treat VSMCs for 24 hours according to the previous study (19).

### Ang II induced AAA model construction

All the protocols for animal experiments conformed to the regulations and guidelines of Shanghai Ninth People’s Hospital, Shanghai Jiao Tong University School of Medicine institutional animal care and done in accordance with the Association for Assessment and Accreditation of Laboratory Animal Care (AAALAC) and the Institutional Animal Care and Use Committee (IACUC) guidelines. 12-week-old ApoE^-/-^ knockout C57BL/6 mice were purchased from Shanghai Research Center for Model Organism and fed with high fat diet (HFD) under athogen-free conditions with a 12h dark/12h light cycle. As described in our previous study (5), the AAA was induced by implanting osmotic minipumps (#Alzet Model 2004, Charles River Laboratories, Inc). In detail, the mice were randomly allocated into groups and anesthetized by peritoneal injection with pentobarbital sodium (50 mg/kg; Boster, Wuhan, China). Then the mice were implanted minipumps containing saline or Ang II (#A9525; Sigma) at a rate of 1000 ng/kg under the skin of the back for 28 days.

### Abdominal aorta tissue collection and histological analysis

When 16 weeks old, the mice were anesthetized by peritoneal injection of 50 mg/kg pentobarbital sodium and infused with 4% polyformaldehyde (PFA) through the left apex of the heart. After harvesting the whole abdominal aorta followed by the subsequent paraffin embedding and sectioning, histological staining was performed. For the immunofluorescence staining, the samples were incubated with FDX1 (bs-11426R, Bioss) and NLRP3 (ab188865, Abcam) antibodies at 4°C overnight. Consequently, the sections were incubated with secondary antibodies for 1h under room temperature. Image J software (Rawak Software, Inc. Germany) was then utilized for quantitative analysis of immunofluorescence intensity as described in our previous study (5).

### RNA extraction and high-throughput RNA sequencing

Abdominal aorta was perfused by saline through the left apex of heart and washed by PBS before RNA extraction. In total, six samples (three from the saline group and three from the Ang II group) were subjected for high-throughput RNA sequencing. For each sample, three random murine abdominal aorta tissues in the same group were mixed into one sample before RNA extraction. Total RNA was extracted using the miRVana miRNA Isolation Kit (Ambion, Texas, USA) following the manufacturer’s protocol. Firstly, Cutadapt was used to remove the reads that contained adaptor contamination (20), low quality bases and undetermined bases. Then sequence quality was verified using FastQC (http://www.bioinformatics.babraham.ac.uk/projects/fastqc/). We used Bowtie2 (21) and Hisat2 (22) to map reads to the genome of AAA tissues. The mapped reads of each sample were assembled using StringTie (23). Then, all transcriptomes from AAA samples were merged to reconstruct a comprehensive transcriptome using perl scripts. After the final transcriptome was generated, StringTie and edgeR was used to estimate the expression levels of all transcripts (24).

### Raw data processing

Cutadapt was utilized to remove the reads that contained adaptor contamination, low quality bases and undetermined bases. Then sequence quality was verified using FastQC (http://www.bioinformatics.babraham.ac.uk/projects/fastqc/). We then used Bowtie2 and Hisat2 to map reads to the genome of AAA samples. The mapped reads of each sample were assembled using StringTie. Then, all transcriptomes from AAA samples were merged to reconstruct a comprehensive transcriptome using perl scripts. StringTie was used to perform expression level for mRNAs and lncRNAs by calculating FPKM.

### Bioinformatics analysis

Firstly, transcripts that overlapped with known mRNAs and transcripts shorter than 200 bp were discarded. Then we utilized Coding Potential Calculator (CPC) (25) and Coding-Non-Coding Index (CNCI) (26) to predict transcripts with coding potential. All transcripts with CPC score <-1 and CNCI score <0 were removed. The remaining transcripts were considered as lncRNAs. StringTie was used to perform expression level for mRNAs and lncRNAs by calculating FPKM (27).The differentially expressed mRNAs and lncRNAs were selected with log2 (fold change) >1 or log2 (fold change) <-1 and with statistical significance (q value < 0.05) by R package – edgeR. To explore the function of lncRNAs, we predicted the cis-target genes of lncRNAs. LncRNAs may play a cis role acting on neighboring target genes. In this study, coding genes in 100,000 upstream and downstream were selected by python script. Then, we showed functional analysis of the target genes for lncRNAs by using the BLAST2GO (28). Significance was expressed as a p value < 0.05.

### Quantitative real-time PCR

To validate the reliability of the RNA sequencing results, we randomly selected and analyzed 5 DEmRNAs using qRT-PCR analysis. Total RNA was extracted using the TRIzol reagent and reversed transcribed into cDNA using random primers. Quantification of the LncRNA and mRNA was performed using a Sequence Detection System. Three independent experiments were conducted for each sample. The primer sequences presented in **Table 1** were synthesized by Shanghai Sangon Biotech Co., Ltd. (Shanghai, China).

**Table 1:** Primer nucleotide sequences of RT-qPCR.

Name	Primer sequence
mus-myh1-F mus-myh1-R	5’- GACTACAACATCGCTGGCTG -3’ 5’- CTTGGCCCCTTTCTTTCCAC-3’
m-Ttn-F	5’-GATCATTGTCCCTGCGTCAC-3’
m-Ttn-R	5’-TCATTTCGAGCCTGGAACCT-3’
m-Ryr1-F	5’-TGGAGATCACAGCCCACAAT-3’
m-Ryr1-R	5’-CAGATGAAAGGATGGTGCGG-3’
m-FDX1-F	5’-CGTTGGCTTGCTCTACTTGT-3’
m-FDX1-R	5’-GCTGGGCTGAAGGAAATAGG-3’
m-NLRP3-F	5’-TCTCCCGCATCTCCATTTGT-3’
m-NLRP3-R	5’-CTGTCCCGCATTTTAGTCCG-3’
m-β-actin-F	5’-CACGATGGAGGGGCCGGACTCATC-3’
m-β-actin -R	5’- TAAAGACCTCTATGCCAACACAGT -3

### Western blot

Total AAA tissue or cell samples were lysed using RIPA peptide lysis bufer containing 1% protease inhibitors (Roche; #11836153001). After being sealed with 5% bovine serum albumin (BSA) (#B2064, Sigma), the membranes were incubated with the following antibodies: anti‐FDX1(#12592-1-AP, Proteintech), anti‐NLRP3 (#27458-1-AP, Proteintech), anti-GAPDH (glyceraldehyde‐3phosphate dehydrogenase) at 37°C overnight. Then we rinsed the membranes were three times with tris-buffered saline tween (TBST). Subsequently, the rinsed membranes were incubated with secondary antibodies (#7074, CST) at room temperature for 2h. We examined and visualized the intensity of protein signals by an enhanced chemiluminescence (ECL) reagent (#GERPN2106, Sigma) and a Biorad Gel Doc EQ system. And the grey value was analyzed using Image J software.

### Functional and pathway enrichment analyses

GO and KEGG pathway analyses were conducted through the OmicStudio tools at https://www.omicstudio.cn/tool to predict the potential functions of DE mRNAs and DE lncRNAs. KEGG pathway analysis was conducted to predict the involvement of differentially expressed genes in the biological pathways. The top 10 enriched GO terms and top 20 enriched pathways were ranked by enrichment score (-log10(p-value)) identified by the database for annotation, Visualization, and Integrated Discovery.

### The expression of CRGs in AAA and normal samples

As indicated previously (10),18 genes were demonstrated to be associated with cuprotosis. In order to confirm the involvement of these CRGs in AAA, we compared the expression patterns of these 18 CRGs between AAA and normal tissues.

## Results

### The expression profile of lncRNA and mRNA in the AAA model

**Figure 2:**
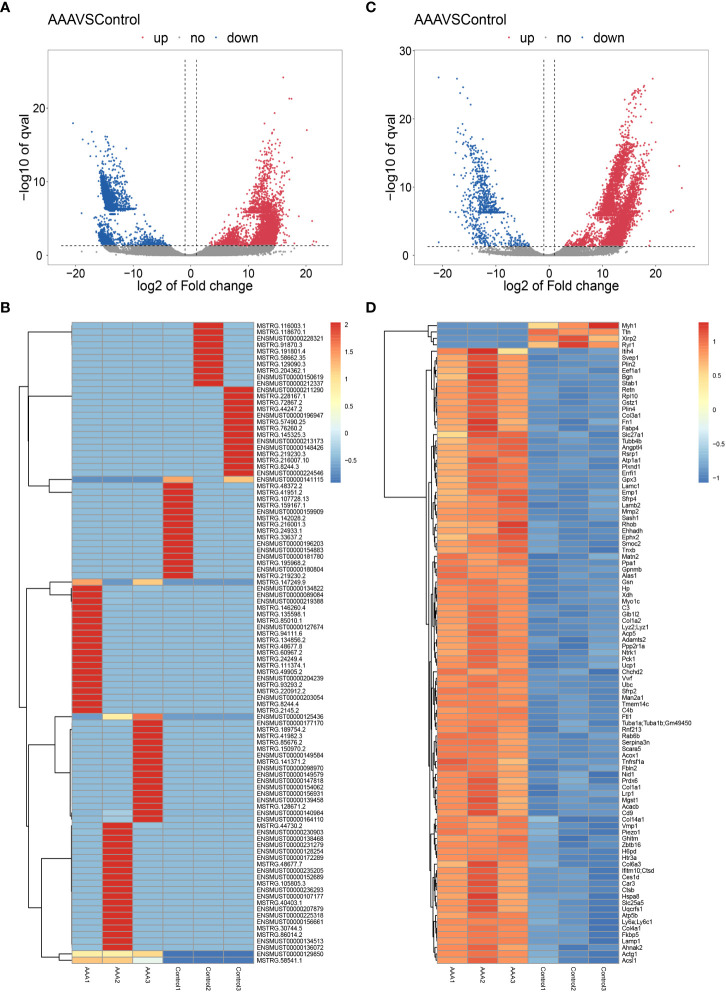
Expression profiles of LncRNAs and mRNAs in mouse after AAA. Scatter-plot for comparing global expression profiles of lncRNAs **(A)** and mRNAs **(C)** in the artery between the AAA and sham-operated mice. X and Y axes indicate the mean normalized signal values (log2 scaled). Blue points represent up-regulated lncRNAs or mRNAs while red points represent downregulated lncRNAs or mRNAs. The heat map shows hierarchical clustering of DElncRNAs **(B)** and DEmRNAs **(D)** in the artery between the AAA and sham-operated mice. The color scale indicates the expression of DElncRNAs and DEmRNAs.

**Figure 3:**
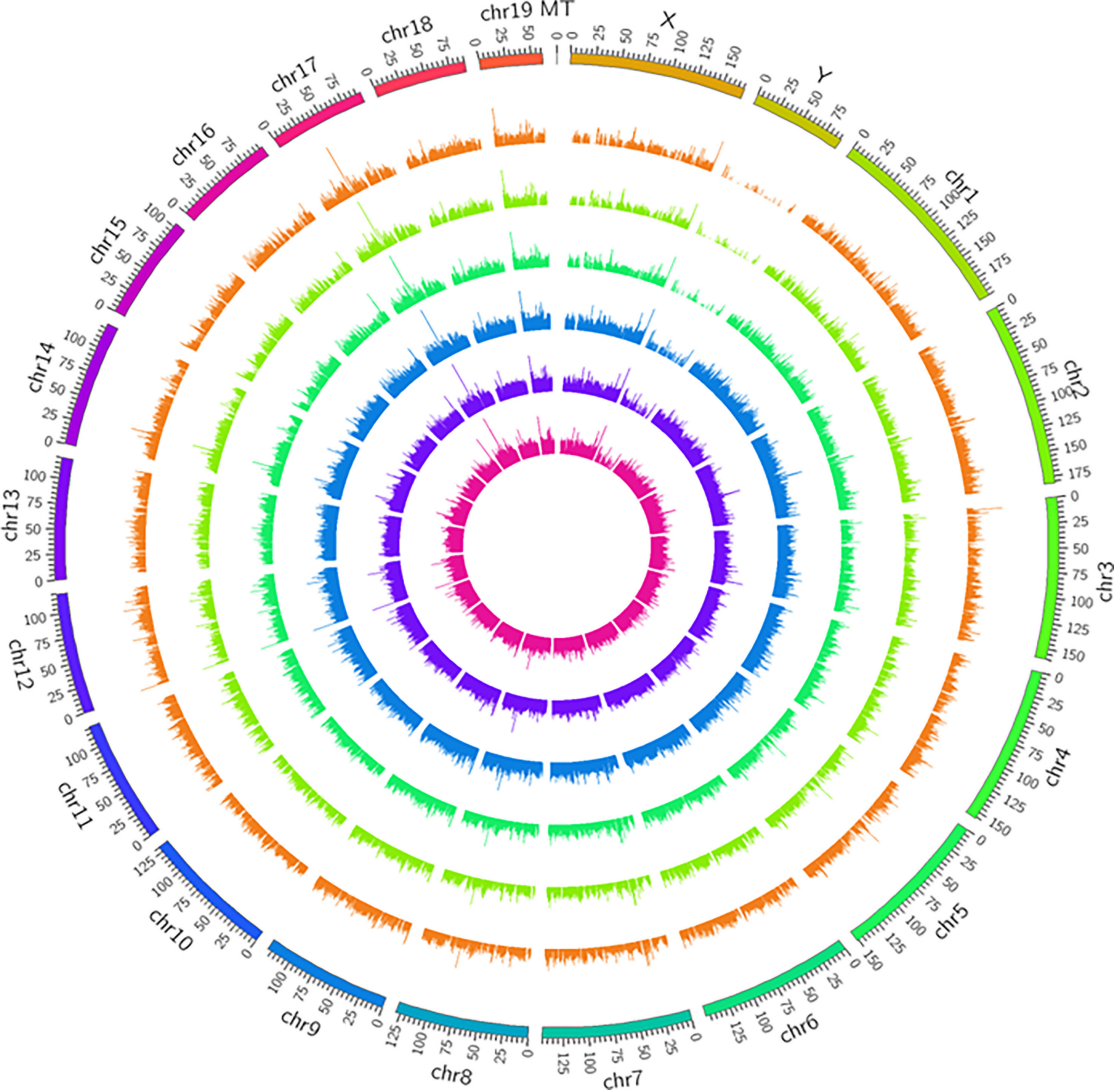
Circos plots representing the distribution of DElncRNAs and DEmRNAs on mice chromosomes. The outermost layer of the circos plot is the chromosome map of the rat genome. The largest and larger inner circles represent all DElncRNAs detected by RNA-sequencing with fold change ≥2.0, p < 0:05, and FDR < 0.05. The increased or decreased lncRNAs are marked with red or green bars, respectively, and bar heights in the larger inner circle indicate numbers of DElncRNAs. The smaller and smallest inner circles represent all DEmRNAs detected by RNA-sequencing with fold change ≥2.0, p < 0:05 and FDR < 0.05. Increased or decreased mRNAs are marked with red or green bars, respectively, and bar heights in the smallest inner circle indicate numbers of DEmRNAs.

In the study, 27616 lncRNAs and 2189 mRNAs were significantly differentially expressed, with fold change ≥2.0, P<0.05. In total, there were 10424 up-regulated and 17192 down-regulated lncRNAs, 1904 up-regulated and 285 down-regulated mRNAs. Scatter plots analyses showed the expression signatures (**Figure 2**). Hierarchical clustering expression showed significant differences in the abdominal aorta tissue between AAA and control mice. Furthermore, transcripts were proved to be distributed on all chromosomes (**Figure 3**).

### Validation of differentially expressed mRNAs in AAA and control group

**Figure 4:**
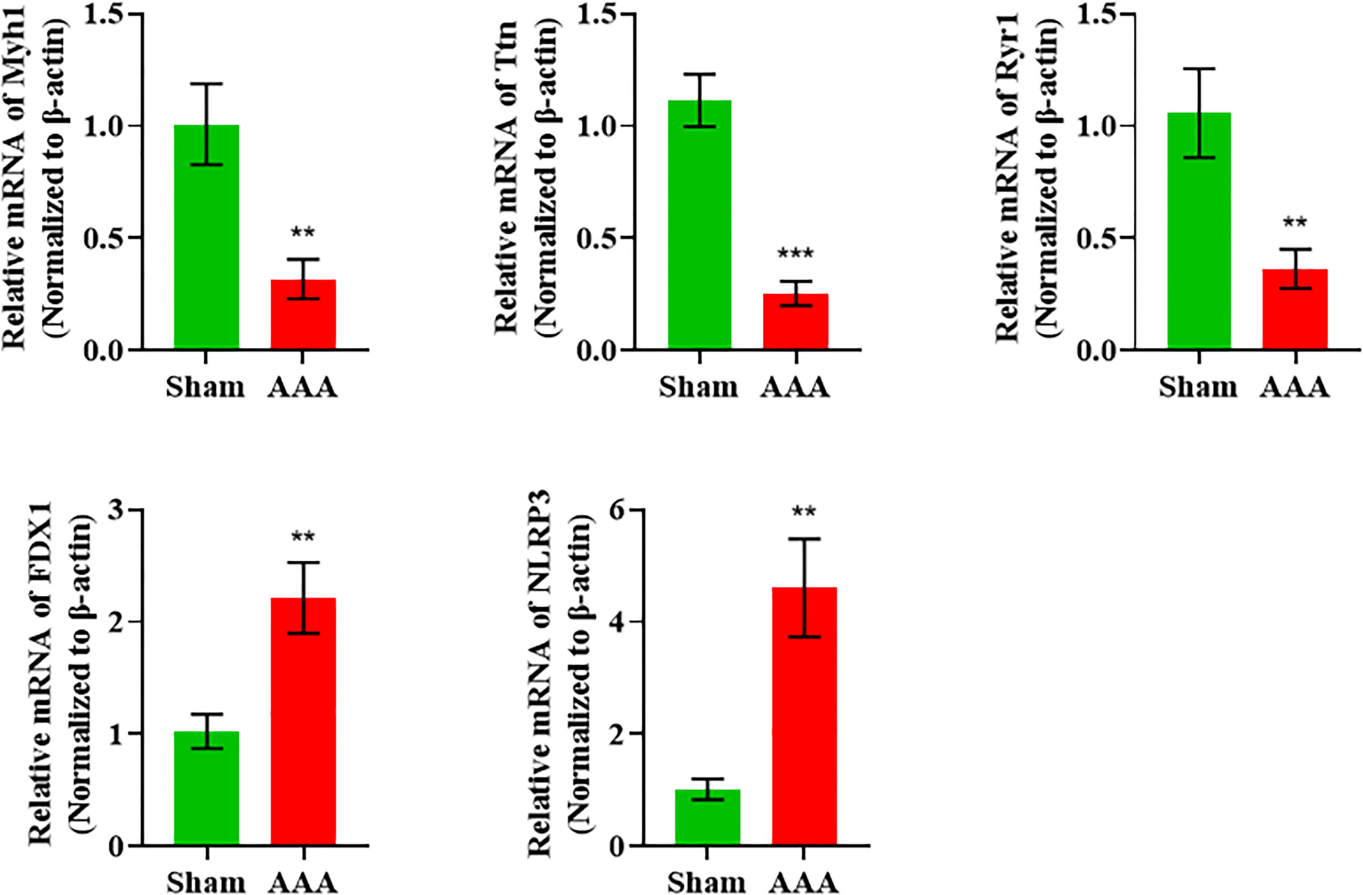
qRT-PCR validation of DElncRNAs and DEmRNAs in the AAA mice compared with matched tissues of sham-operated mice. **p value < 0.01, ***p value < 0.001

As we can see from **Figure 2D**, the Myh1, Ttn and Ryr1 were mostly obvious down-regulated genes, yet they were not cuprotosis-related genes. Thus, in order to validate the cuprotosis-related genes more comprehensively, we used the RT-qPCR to further elucidate the data of RNA sequencing. The mRNAs were randomly selected to verify the high throughput RNA sequencing results in six sample pairs by qRT-PCR. We found that the expression of were up-regulated and were down-regulated in the AAA modes The qRT-PCR results were in line with the high sequencing results (**Figure 4**). Hence, the results validated our data as highly reliable and showed that these mRNAs might be involved in AAA pathogenesis.

### GO and KEGG analyses of differentially expressed genes in the AAA and normal samples

**Figure 5:**
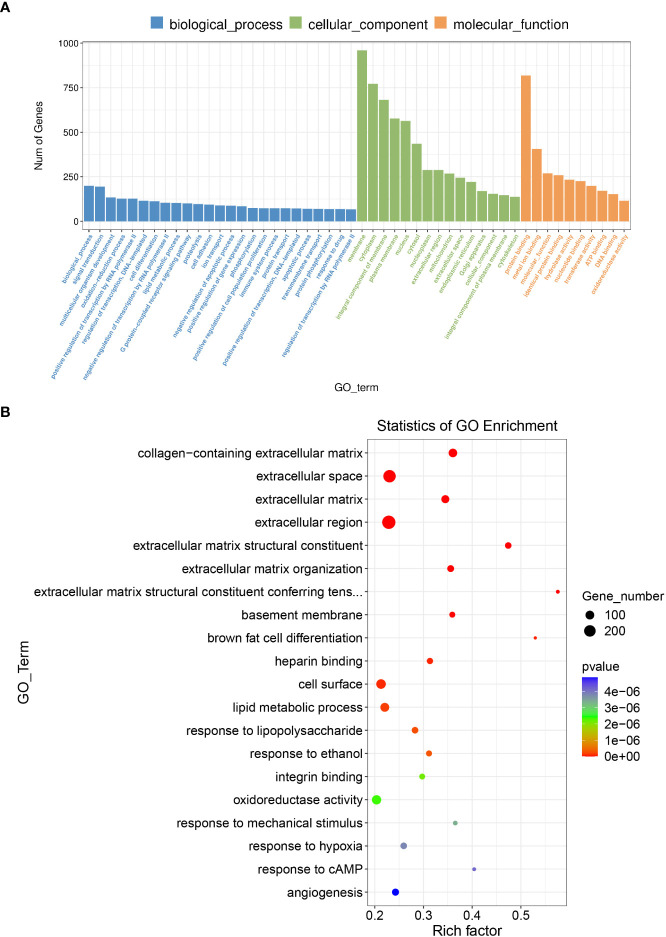
GO enrichment analysis for the DEmRNAs with the 10 highest enrichment scores. **(A)** GO enrichment analysis for co-expressed DEmRNAs of DElncRNAs. Blue bars are biological processes, green bars are cellular components, and yellow bars are molecular functions. **(B)**The size of the spot indicates the gene numbers enriched in the pathway, and the color of the spot indicates the significance level of the enriched pathway.

**Figure 6:**
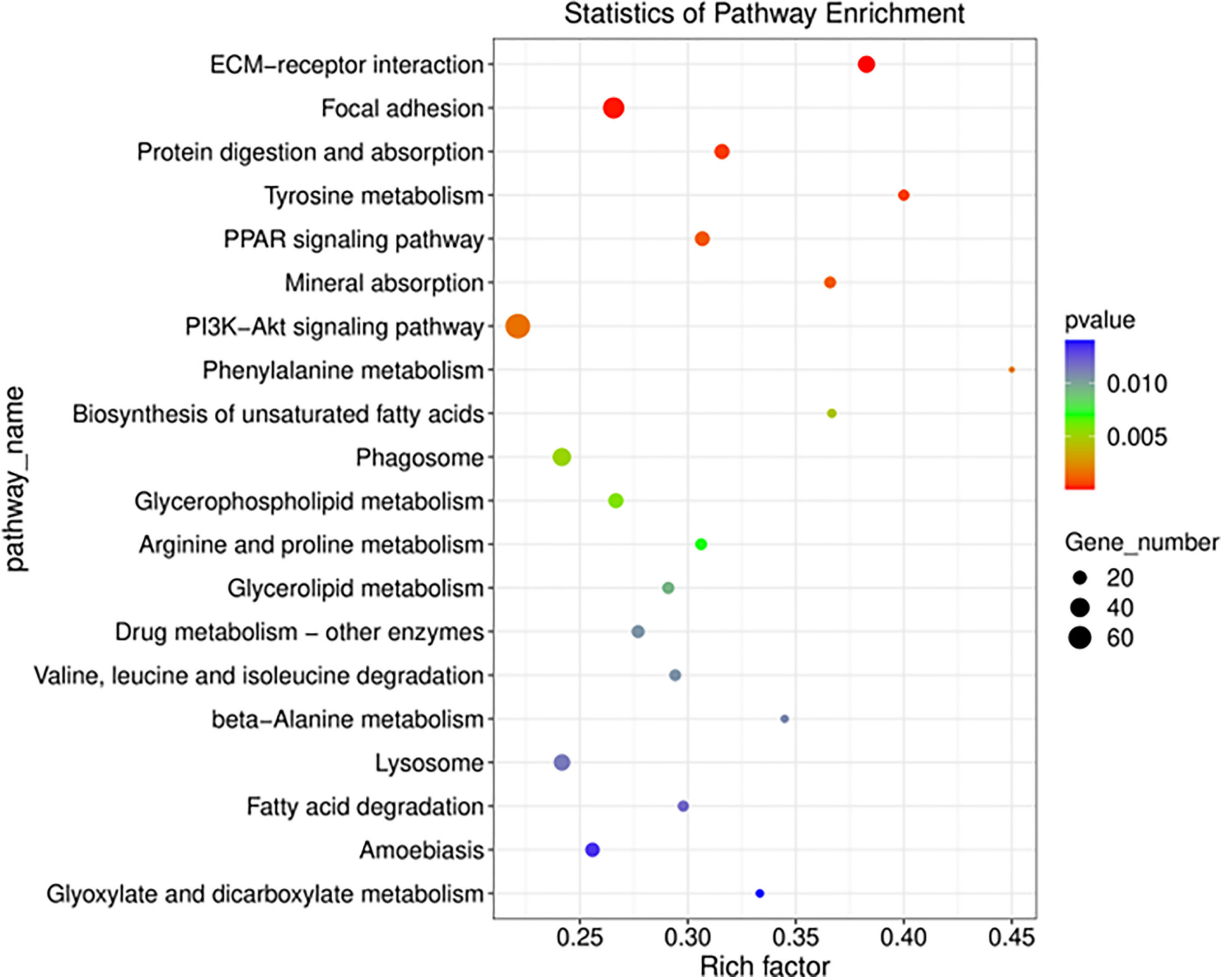
Functional annotation and KEGG pathway enrichment analyses for co-expressed DEmRNAs of DElncRNAs. The size of the spot indicates the gene numbers enriched in the pathway, and the color of the spot indicates the significance level of the enriched pathway. The abscissa is the enrichment score. Size represents the number of enriched genes, and color indicates the degree of enrichment. Higher enrichment scores correlate with lower P-values, indicating that the enrichment of differentially expressed genes in given pathway is significant.

In GO and KEGG pathway enrichment analyses of DEmRNAs, we found 2189 mRNAs that were differentially expressed. GO enrichment analysis showed that the enriched GO biological processes for the differentially expressed genes in the AAA group were regulation of collagen-containing extracellular matrix, extracellular space, extracellular matrix (ECM) and extracellular region (**Figure 5**). Similarly, differentially expressed genes were analyzed using KEGG. We found that the genes in the AAA were involved in peroxisome proliferators-activated receptor (PPAR), phosphatidylinositol-3-hydroxykinase (PI3K)-AKT, ECM-receptor interaction and fatty acid degradation signaling pathways (**Figure 6**), which were demonstrated as critical pathways in immune system (29–31).

### Validation of the expression of crucial cuprotosis-related differentially expressed genes in AAA mice

**Figure 7:**
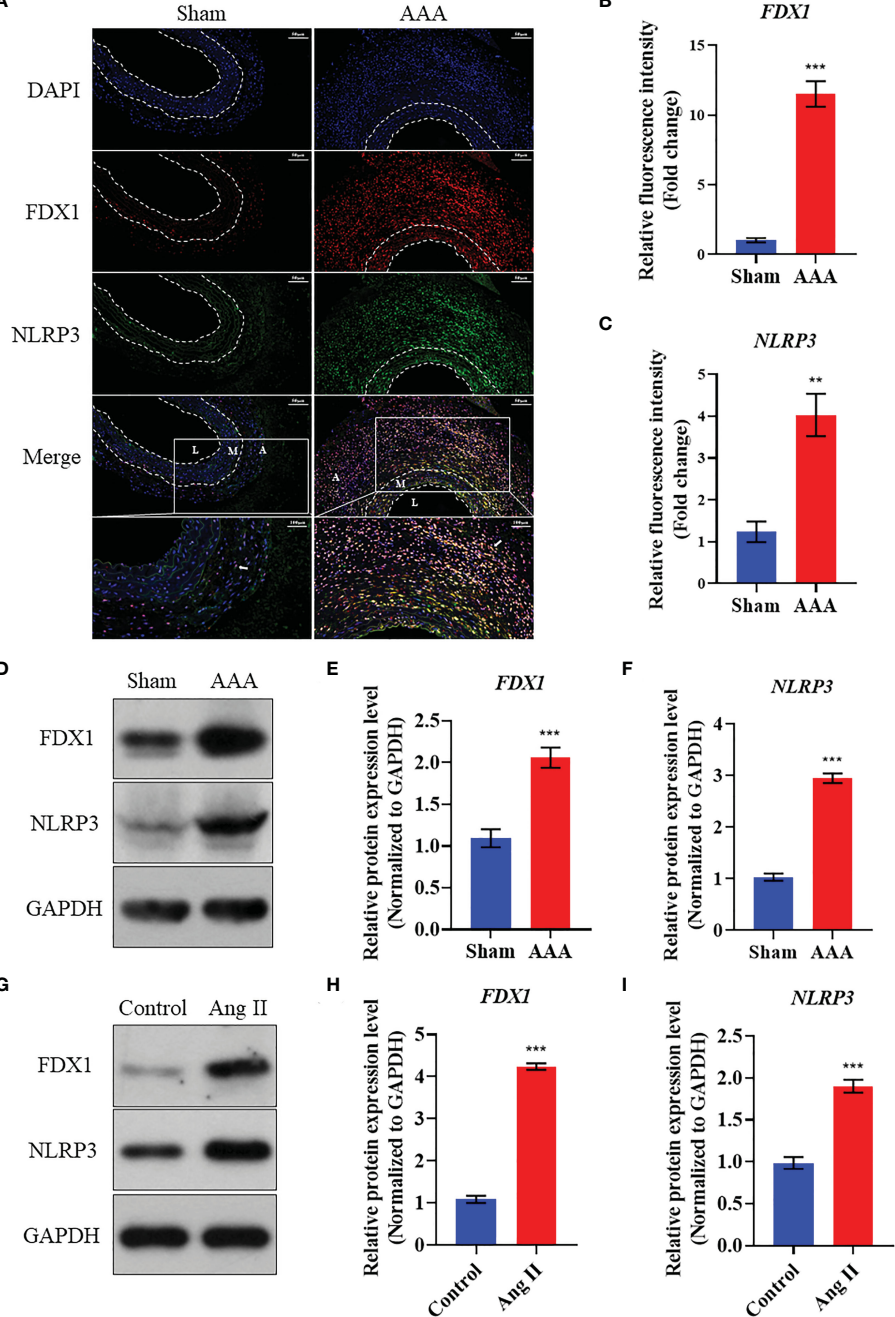
Validation of the expression of FDX1 and NLRP3 in Ang II induced AAA model. **(A)** The immunofluorescence images of FDX1 and NLRP3 on abdominal aorta in Sham group and AAA group. Scale bar, 50 μm and 100 μm. L: Tunica Lumen; M: Tunica Medium; A: Tunica Adventitia. **(B)** Quantitative analysis of FDX1 fluorescence intensity in the two groups. **(C)** Quantitative analysis of NLRP3 fluorescence intensity in the two groups. **(D–F)** Western blot analysis of FDX1 and NLRP3 on abdominal aorta in Sham group and AAA group. **(G–I)** Western blot analysis of FDX1 and NLRP3 on VSMCs in Control group and Ang II group. Repetition=3, **p value < 0.01, ***p value < 0.001.

As **Figures 7A–C** showed, FDX1 expression level increased in the AAA tissues compared to the abdominal aortic walls of sham group in ApoE^-/-^ mice model. Meanwhile, the immunofluorescence analysis revealed that NLRP3 upregulated in the AAA model to the abdominal aortic walls of sham group. Interestingly, we also found that FDX1 and NLRP3 were co-localized in the AAA tissues, suggesting the functional relationship between FDX1 and NLRP3. In addition, the expression of FDX1 and NLRP3 in AAA samples mainly existed in the adventitia, which played a critical role in the development of AAA (32, 33). Besides, the data of western blot experiment was broadly consistent with that of immunofluorescence analysis (**Figures 7D–F**). Furthermore, the death of VSMCs has been confirmed to promote AAA formation (19, 34), which can be induced by the accumulation of cellular copper ions (16). Thus, we used Ang II to stimulate the VSMCs and we found that FDX1 and NLRP3 elevated in VSMCs after the induction by Ang II, indicating that the cuprotosis of VSMCs was of great significance in the pathogenesis of AAA.

### Expression verification of the CRGs in the AAA specimen

As **Table 2** presented, all these 18 CRGs were identified to be differentially expressed between AAA and normal samples. Taken together, we found that 13 genes were upregulated in while 5 genes were downregulated in AAA tissues, which might be novel targets in the treatment of AAA patients. Then, pathway enrichment analysis with these CRGs was performed. The most enriched KEGG pathways were the C-type lectin receptor, necroptosis and NOD-like receptor signaling pathways.

**Table 2:** The expression of cuprotosis related genes in abdominal aortic aneurysm specimen.

Genes	Log2(fc)	q value	regulation
NFE212	0.48	0.02	UP
NLRP3	5.91	0.04	UP
ATP7b	0.68	0.86	UP
ATP7a	0.63	0.00	UP
LIPT1	0.43	0.88	UP
FDX1	0.60	0.19	UP
DLST	0.54	0.00	UP
DBT	0.30	0.05	UP
MTF1	0.54	0.02	UP
GCSH	0.50	0.45	UP
DLD	0.10	0.14	UP
DLAT	0.28	0.00	UP
PDHA1	0.25	0.00	UP
LIPT2	-0.67	0.91	DOWN
SLC31A1	-0.04	0.06	DOWN
PDHB	-0.09	0.34	DOWN
LIAS	-0.13	0.89	DOWN
GLS	-0.52	0.08	DOWN

## Discussion

In this present study, we identified global expression changes of lncRNAs and possible relationships with coding genes in the delayed phase of AAA for the first time. A sum of 10424 up-regulated and 17192 down-regulated lncRNAs were found to be significantly differentially expressed in the AAA model. Accordingly, 1904 up-regulated and 285 down-regulated mRNAs were identified in the AAA model, suggesting that they were likely to be involved in pathological processes. Subsequently, five mRNAs were chosen for qRT-PCR validation, and the qRT-PCR results were in accordance with high throughput sequencing data. GO and KEGG pathway enrichment analyses revealed the role of differentially expressed mRNAs in AAA pathogenesis.

Cuprotosis is a novel cell death pattern characterized by the accumulation of intracellular free copper and protein lipidation causing cytotoxic stress, which ultimately results in cell death (35). In multiple organisms, copper is an essential metal element and transition factor with redox activity, involved in the regulation of several physiological processes like mitochondrial respiration, iron uptake, energy metabolism and antioxidation (36). Under physiological status, copper ions acts as cofactors to maintain a dynamic balance and cell homeostasis through the direct binding to multiple proteins or enzymes. Whereas, copper ions can induce a proteotoxic stress response under internal environment disorders and pathological conditions by disturbing the fatty acylated component homeostasis of the Krebs cycle, leading to the aggregation of fatty acylated proteins (37). Multiple studies have demonstrated that copper metabolism is correlated with the pathogenesis of various cardiovascular diseases, such as atherosclerosis, coronary heart disease, and cardiac hypertrophy (38–40). Multiple studies have confirmed that reactive oxygen species (ROS) play a crucial role in the pathogenesis of AAA (41). In particular, elevated ROS levels in macrophages can lead to the progression of AAA (42). Besides, the available clinical evidence suggests that circulating serum samples from AAA patients displayed a tendency of copper-induced low-density lipoprotein oxidation and vascular smooth muscle cells ROS production (43). The accumulation of copper in vascular smooth muscle cells will lead to the cell death of vascular smooth muscle cells, that is, cuprotosis (44, 45).

Cuprotosis is closely correlated with a variety of pathogenesis such as apoptosis and inflammation in vascular endothelial cells, smooth muscle cells and macrophages (39), involved in the changes of each vascular layer in the pathological changes of AAA immune environment. For example, the copper transporter ATP7A played a critical role in PDGF-induced vascular smooth muscle cell migration (46). And it has been reported that ATP7A can limit vascular inflammation and AAA development by regulating miR-125b (16). ATP7A also exists as a modulator of oxidative response in vascular smooth muscle cells (47). Besides, copper ions induced oxidative DNA damage and cell death *via* copper ion-mediated p38 MAPK activation in vascular endothelial cells, mainly found in the intima of vessels (48). In this study, we found that CRGs were correlated with inflammatory response and oxidative response, including NLRP3 and FDX1. In our previous study, macrophage NLRP3 inflammasome plays a significant role in promoting the progress of AAA (5). Furthermore, NLRP3 inflammasome activation regulates vascular smooth muscle cells phenotypic switch (49), which ultimately leads to AAA development through tunica medium elastin degradation (4). However, few studies have demonstrated the role and potential mechanism of FDX1 in AAA formation, which may become a promising research orientation.

The traditional view is that vascular wall inflammation involves an ‘inside-out’ response, which begins with endothelial cell activation and leukocyte extravasation, and progresses from the inside-out to the adventitia (3). Recently, there is a novel concept that the inflammatory infiltration and matrix remodeling of the outer membrane occurs earlier than the changes of intima in the early course of human AAA, which indicates the rationality of ‘outside to inside’ theory (50). In addition to adventitia macrophages, T lymphocytes are another major immune cell subset in human AAA adventitia. And the infiltration of adventitia T lymphocytes is strongly related to AAA diameter, which is also an important indicator of AAA expansion (51). It has been shown that adventitia T lymphocyte-derived EVs accelerate AAA development by driving macrophage redox imbalance and migration (3). Besides, perivascular adipose tissue (PVAT) is distributed around the abdominal aortic adventitia and is in direct contact with the adventitia anatomically, in which brown and white adipocytes exist the major cell types (52, 53).

This present study firstly identified and validated NLRP3 as the cuprotosis-related differentially expressed gene in AAA samples, and NLRP3 inflammasome has been reported to trigger the AAA progression by inhibiting the browning of PVAT (54). The browning of PVAT proved to improve the immune microenvironment of adventitia, leading to the blockade of AAA (55–57). In addition, researchers have revealed that NLRP3 inflammasome induces CD4+ T cell loss in pathological condition (58). To our knowledge, dysregulation of intracellular copper homeostasis in macrophages, T lymphocyte and adipocytes is widely observed during various disorders (59–61). However, how cuprotosis in adventitia macrophages, T lymphocyte and adipocytes are involved in the occurrence of AAA remains unsolved.

FDX1, a key gene for cuprotosis, is the upstream regulator of protein lipid acylation, plays significant role in immune microenvironment infiltration (62). Several studies have reported that protein lipid acylation is relevant to the metabolic dysfunction of fatty acids, which contributes to the incidence of cardiovascular diseases including atherosclerosis and AAA (63–65). Furthermore, fatty acids-derived acetyl-CoA not only fuels the tricarboxylic acid (TCA) cycle, but also causes non-enzymatic mitochondrial protein hyperacetylation, thus impairing complex I activity and mitochondrial ROS production (66). Nevertheless, there is no specific research report on the relationship between FDX1 expression and the outcome of AAA. Hence, a fundamental core mechanism underlying AAA immune environment infiltration and FDX1 is required to be uncovered. Herein, we attempted to provide insight into the metabolic pattern of FDX1 in AAA immune environment. We found that FDX1 expressed higher in AAA samples compared to the abdominal aortic walls in sham group, coupled with co-localization with NLRP3, suggesting the potential connection between the two key genes of cuprotosis in AAA.

NOD-like receptor protein 3 (NLRP3) inflammasome, a potential therapeutic target for a variety of cardiovascular diseases, can sense pathogen-related molecular patterns and endogenous danger signals (67, 68). Our previous work demonstrated that exosomes from adipose-derived mesenchymal stem cells inhibit AAA chronic inflammation by the blockade TXNIP-NLRP3 inflammasome (5). What is noteworthy, NLRP3/IL-1β signaling pathway can be used to promote vascular adventitia remodeling in the case of PVAT dysfunction (69). In addition, researchers have reported that the upregulation of NLRP3 due to autophagy defect can induce brown fat dysfunction in mice (70). Interestingly, NLRP3 knockout of macrophages in mouse models can reduce the production of IL-1β thereby inhibiting the browning of PVAT. These results suggest that downregulation of NLRP3 in macrophages can also promote the browning of PVAT, regulate the inflammatory microenvironment of PVAT and metabolic reprogramming (54). In this current study, we also revealed that the elevation of NLRP3 mainly existed in the adventitia of AAA tissues, which will be further explored in the following studies. As the first study to reveal the biological significance of FDX1 and NLRP3, two key proteins of copper death, in AAA development, our experimental results in **Figure 7** showed that the expression of FDX1 and NLRP3 increased in AAA with a high degree of consistency. The colocalization of these two genes suggested a potential relationship between the two genes’ functions. The existing literature has not confirmed the direct relationship between FDX1 and NLRP3, but researchers suggested that ATP7A and NLRP3 are negatively correlated in pan-cancer (71). As is known to all, ATP7A is a copper ion transporter, which plays an important role in the accumulation of copper ions in cells (72). Importantly, FDX1 has recently been shown to be a key protein in copper death (73). We will also further verify the underlying mechanism between FDX1 and NLRP3 by knocking out FDX1 in our future experiments.

However, it should be noted that this study still had some limitations. Firstly, the number of animals included in each group for RNA sequencing might be relatively small to identify differentially expressed genes, which would be further expanded in our future studies. Besides, clinical specimen from AAA patients needed to be added in to further elucidate the FDX1 and NLRP3 expression. Finally, And further experiments are needed to reveal the potential mechanisms of CRGs in AAA formation. For instance, we will construct FDX1 smooth muscle cell specific knockout mice in subsequent experiments to explore the potential mechanism of these two genes for AAA pathogenesis.

## Conclusion

Taken together, we conducted RNA sequencing using abdominal aorta tissues from AAA and control group and found that 27616 lncRNAs and 2189 mRNAs were significantly differentially expressed. Moreover, 18 CRGs (thirteen up-regulated genes and five down-regulated genes) were obtained, of which FDX1 and NLRP3 were screened out for further validation using immunofluorescence analysis. Finally, the results of KEGG pathway enrichment analysis revealed that these two CRGs were enriched in the C-type lectin receptor, necroptosis and NOD-like receptor signaling pathways, suggesting the potential role of FDX1 and NLRP3 in the immune environment of AAA. As far as we know, this is the first research to uncover the cuprotosis related genes in AAA development, thus offering a novel insight to the therapy of AAA patients.

## Data availability statement

The datasets presented in this study can be found in online repositories. The names of the repository/repositories and accession number(s) can be found below:PRJNA922712 (SRA).

## Ethics statement

The animal studies were performed in compliance with the regulations and guidelines of Shanghai Ninth People’s Hospital, Shanghai Jiao Tong University School of Medicine institutional animal care.

## Author contributions

JH, SX, and ZX contributed equally to this article. XBL, BL, and XWL designed the experiments. JH and SX performed the experiments. JH, SX, and ZX drafted the article. ZW and XX participated in the AAA animal model construction. XW and GL take the responsibility of language correction. All authors contributed to the article and approved the submitted version.
